# The Impact of Australian Bushfires on Asthma Medicine Prescription Dispensing

**DOI:** 10.3390/healthcare12040428

**Published:** 2024-02-07

**Authors:** Zhihua Zhu, Mark Naunton, Reza Mortazavi, Mary Bushell

**Affiliations:** Discipline of Pharmacy, School of Health Sciences, Faculty of Health, University of Canberra, Canberra, ACT 2617, Australia; lindazhihuazhu@gmail.com (Z.Z.); mark.naunton@canberra.edu.au (M.N.); mary.bushell@canberra.edu.au (M.B.)

**Keywords:** wildfire, bushfire, asthma, air pollution, PBS, PM_2.5_, PM2.5, smoke, prescriptions, medicines

## Abstract

Background: Air pollution can cause numerous health problems and increase the need for medicines to treat and prevent asthma in affected areas. There is limited evidence about the association between airborne particles with a diameter of 2.5 micrometres or smaller (PM_2.5_) and asthma medicine usage. This study examined the potential association between the levels of PM_2.5_ and the supply of prescription asthma medicines in the Australian Capital Territory (ACT), Australia, during the severe bushfire season between November 2019–January 2020. Methods: Daily data was obtained from an ACT air quality monitoring station from November 2019 to January 2020 (study period) and November 2018 to January 2019 (control period, no bushfire). The number and types of government-funded asthma medicine prescriptions were obtained from the Services Australia (government) website by searching under ‘Pharmaceutical Benefits Scheme Item Reports’ and using relevant item codes during the study and control periods. Results: The medians for PM_2.5_ levels for the study period were significantly higher than those for the control period (*p* < 0.001). There were increases in the number of dispensed prescriptions of short-acting beta-2 agonists (SABA), inhaled corticosteroids, and long-acting beta-2 agonists combined with inhaled corticosteroids. The greatest difference was seen with the inhaled corticosteroids: a 138% increase. Conclusions: The increase in the number of dispensed asthma prescriptions during the bushfire season should be used to inform the stock holdings of these medicines in preparation for future events to ensure access to lifesaving asthma medicines.

## 1. Introduction

Australia reports some of the best air qualities in the world. Despite this, it is vulnerable to temporary spikes of extreme air pollution due to dust storms and devastating bushfires (wildfires). During a bushfire season, the air quality can deteriorate enormously within and around the bushfire affected area [1].

It is estimated that almost 2600 Australians die from air pollution annually [2]. Reports show a positive association between air pollution caused by bushfires and increased exacerbations, hospital admissions or emergency department attendances due to respiratory illnesses such as asthma and chronic obstructive pulmonary disease [3,4,5]. During the 2019–2020 summer in Australia (Black Summer), many people on the East Coast of this country experienced an extreme bushfire season [6]. More than 17 million hectares of bushland was destroyed by fire [7]. During this season, the thick smoke haze was visible in Canberra (Australia’s capital city) which made it one of the most polluted cities in the world. This was evidenced by its Air Quality Index (AQI), which is calculated based on rolling average concentrations of floating particles in the air (such as particles with a diameter of 2.5 micrometres or smaller (PM_2.5_), particles with a diameter of 10 micrometres or smaller (PM_10_), and concentrations of gaseous pollutants in the air including nitrogen dioxide (NO_2_), sulfur dioxide (SO_2_), carbon monoxide (CO), as well as the ground-level ozone concentration) [1,8,9,10]. The concentrations of airborne particulate materials (PM_2.5_ and PM_10_) increase in bushfires [11]. The PM_2.5_ particles are small enough to deposit in human bronchioles and alveoli, which can then induce oxidative stress and inflammation in the lung tissue. Due to the minuscule size of these particles, they can enter the bloodstream and be carried to other tissues [12]. PM_2.5_ poses the greatest risk to health and is considered a very dangerous category of particles [13].

On New Year’s Day 2020, the AQI reached 5185 at the Monash Air Quality Station in Canberra, twenty-five times higher than the hazardous threshold of 200. This caused significant public concerns about the air quality and respiratory health and led the Australian Capital Territory (ACT) Department of Health to advise people living in the ACT, especially those with pre-existing respiratory conditions such as asthma, to stay indoors and use face masks to minimise the impacts of inhaling smoke [1]. What followed next was a shortage of P2/N95 face masks.

For context, Canberra is the capital and the only city of the Australian Capital Territory. According to the 2021 census, there were 454,000 ACT residents [14]. The total area of the ACT is 2358 km^2^, which is less than 1% of Australia’s total land mass [15]. Of this land mass, over 70% of the ACT is open space and mostly bushland [16]. The overwhelming majority of ACT residents live in Canberra. When reporting the population of Canberra, this is usually synonymous with reporting the population of the ACT.

Informed by the above health alert in the ACT, we first conducted a literature review to gather information about the associations between fine particulate matters in the air and respiratory conditions (asthma in particular). A handful of studies had established positive correlations between particulate matters with a median aerodynamic diameter equal to or less than 10 micrometres (PM_10_) arising from bushfire smoke and incidents of asthma hospitalisations [17], or respiratory hospitalisations in general in Sydney and Brisbane (major cities in Australia) [4,18]. Smoke events caused by bushfire have been associated with increased hospital admissions for respiratory diseases, including asthma, in Sydney, Newcastle, and Wollongong (all in Australia) [19]. Those studies mainly captured patients with respiratory symptoms towards the most severe end of the spectrum. Another study in England (which included patients with less severe symptoms) established that salbutamol (SABA) prescriptions in the primary health care sector increased by 1% (95% CI 0.1–2%) with an increase of 10 μg/m^3^ in ambient PM_10_ levels. However, in that study, the PM_10_ originated from urban sources rather than bushfires [20]. Another study had found a significant positive association between PM_2.5_ from urban sources and the frequency of reliever medicines usage. Nevertheless, this study did not identify evidence for associations between PM_2.5_ and the frequency of asthma controller usage [21]. Furthermore, a study in Southern California concluded that exposure to wildfires is associated with increased medicine use for asthmatic children, but the smoke exposure measurement was mainly subjective and questionnaire-based [22].

As mentioned above, most of the studies that we found in the literature have reported a positive association between fine particulate pollution arising from bushfires and incidences of asthma-related hospitalisations (asthma exacerbations, mostly severe). However, only a few of those studies looked directly at the impact on the number of medicines prescribed for asthma. Also, most studies focused on a broader range of particulate matters (PM_10_), possibly due to the unavailability of PM_2.5_ measurements [19]. To fill these gaps, we aimed to examine the association between PM_2.5_ and the number of prescribed medicines supplied under the government-funded Australian Pharmaceutical Benefits Scheme (PBS) during the bushfire season of November 2019–January 2020 (Black Summer) in the ACT. The PBS subsidises most prescribed medicines to a cost the individuals can afford and is a central pillar of the universal health care in Australia. We hypothesised that there would be an increase in the quantities of asthma prescriptions supplied in the ACT under PBS and Repatriation Pharmaceutical Benefits Scheme (RPBS) as air quality worsened, that is the concentrations of PM_2.5_ in Canberra’s air went up.

## 2. Materials and Methods

### 2.1. Air Quality

The smoke from the Black Summer bushfires was clearly visible in Canberra from November 2019 and it peaked in January 2020 [6]. We, therefore, chose November 2019 to January 2020 as the study period in which the increased PM_2.5_ levels could be attributable to the bushfire smoke. To control for the urban background PM_2.5_ levels we obtained data from November 2018 to January 2019 to serve as a control period for comparison. Selecting the same months prior to the control period also allowed us to account for seasonal variables such as temperature and pollens. Furthermore, selecting and analysing the same months prior to and not post the bushfire, removed the impact that COVID-19 may have had on the data set.

The 24-h rolling averages of PM_2.5_ concentrations were obtained from the ACT Government’s website [23]. Among the three monitoring stations (Civic, Monash, and Florey), we decided to use the Civic station because it is almost geographically located in the centre of Canberra [23]. The 24 h rolling average of PM_2.5_ is the average concentration of PM_2.5_ (measured in µg/m^3^) over a 24 h period leading to any point in time. Therefore, we collected the 12:00 am data points of the dates 2 November 2019 to 1 February 2020. These are the daily average PM_2.5_ concentrations for the period 1 November 2019 to 31 January 2020. The data points for the control period were collected in a similar way.

### 2.2. Pharmaceutical Benefits Sscheme (PBS) Medicines

We obtained the quantities of asthma prescriptions dispensed from the Services Australia website under ‘PBS Item Reports’. We searched using the associated PBS item codes. The obtained statistics represent the prescriptions supplied to patients in ACT pharmacies under both PBS and RPBS for the study and control periods [24]. Table 1 shows a list of common asthma medications according to the Therapeutic Guidelines website with the corresponding codes that were included in this study to enable the dispensed prescription count.

### 2.3. Statistical Analyses

Data were analysed using SPSS software version 26 (IBM SPSS Statistics for Windows, version 26, IBM Corp., Armonk, NY, USA). The Wilcoxon rank sum test was used to determine whether there was a significant difference in PM_2.5_ medians between the bushfire and control periods. A *p*-value of less than 0.05 was considered statistically significant. If the result was statistically significant, we considered that month to be impacted by bushfire related PM_2.5_. Prescription counts for each asthma medicine were then compared to the monthly mean PM_2.5_ (μg/m^3^) to determine trends. The control and bushfire affected periods were then compared.

## 3. Results

**Figure 1:**
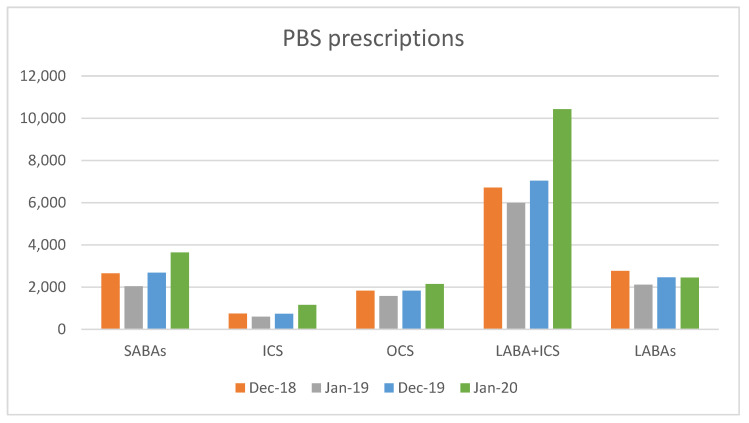
The number of prescriptions for different medication groups over selected months. SABA: short-acting beta agonist; ICS: inhaled corticosteroids; OCS: oral corticosteroids; LABA: long-acting beta agonists.

Figure 1 shows the number of prescriptions for different medication groups over the study periods. The data showed an increase in the prescription numbers for short-acting beta agonist (SABA) and long-acting beta agonists plus inhaled corticosteroids groups (LABA + ICS) in the bushfire affected January 2020.

Table 2 contains a summary of the average values for PM_2.5_ (µg/m^3^) in Canberra and the number of dispensed asthma prescriptions over the study and control periods.

**Figure 2:**
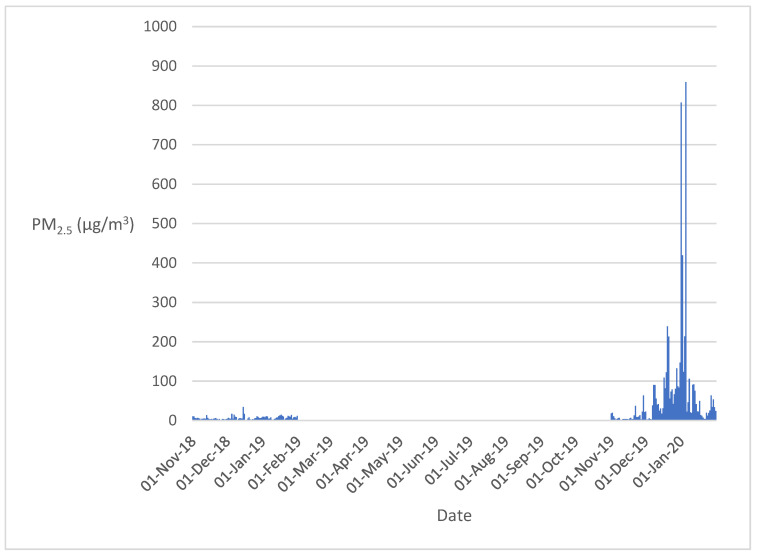
Daily average PM_2.5_ levels over the control (November 2018–January 2019) and bushfire affected (November 2019–January 2020) times.

Figure 2 compares the average PM_2.5_ levels between the study and control periods. There were three missing PM_2.5_ data points for 10–12 December 2018, which was due to a “temporary equipment shutdown” or “data were not measured at the civic station”.

Table 3 provides the data on the total number of prescriptions by drug over the control and test (bushfire affected) periods. The percentage increase is provided.

**Figure 3:**
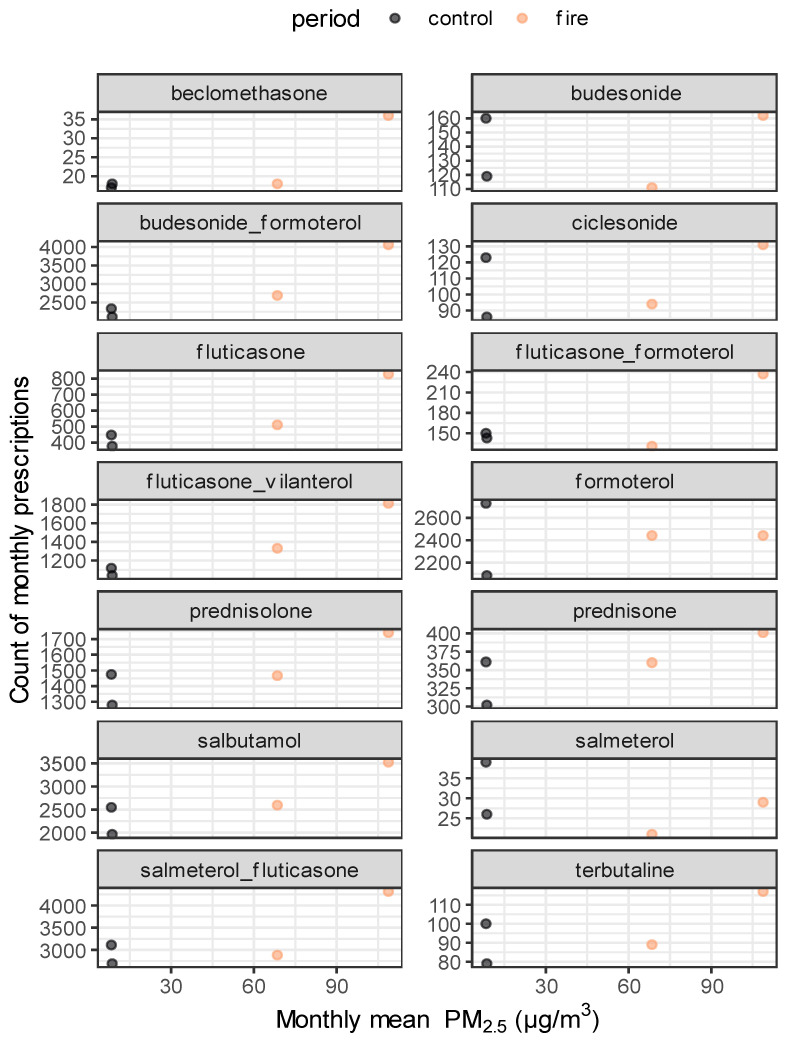
Monthly dispensed asthma prescription count vs. monthly PM_2.5_ levels (µg/m^3^).

There were net increases in the number of dispensed prescriptions for SABA, ICS, OCS, and LABA + ICS prescriptions in the study period compared to the control period. Figure 3 shows the number of dispensed prescriptions for different drug classes for the study period vs. control period and air quality: PM_2.5_ concentrations.

For the control period, there was one day when PM_2.5_ exceeded the national air quality standard of 25 µg/m^3^ [26]. In comparison, there were 43 days when PM_2.5_ values exceeded 25 µg/m^3^ during the test (bushfire) period.

There were only two days in November 2019 in which the PM_2.5_ exceeded 25 µg/m^3^, including 37.9 µg/m^3^ on 22 November and 64 µg/m^3^ on 29 November. The later increase in PM_2.5_ concentrations resulted from smoke plumes and haze travelling to the ACT from the adjacent state’s, New South Wales (NSW), bushfires. In November 2019, no precautionary fire burning was reported in the ACT. Therefore, it is likely that the high PM_2.5_ values measured can be attributed to the ongoing and worsening bushfires at the time [27]. During the Black Summer period, the highest measured PM_2.5_ concentration was 859 µg/m^3^ on 5 January 2020, again due to the transfer of heavy smoke from the NSW bushfires to the ACT [28]. There was a total of 41 days in Dec 2019 and Jan 2020 combined where PM_2.5_ measurements exceeded 25 µg/m^3^.

The data shows that worsening air quality increased the number of asthma prescriptions dispensed across all relevant drug classes (SABAs, LABAs, OCS, SABAs + ICS, and ICS). This includes relievers, preventers, and combination products. It should be noted that when looking at individual active ingredients, inconsistent with all other active ingredients, salmeterol and budesonide were prescribed fewer times during the bushfire affected period when compared to the control period. 

The greatest increase in dispensed prescriptions was for the combined preventer and reliever inhalers. All combination inhalers were dispensed more frequently during the bushfire season compared to the control season. For example, the budesonide and formoterol combination inhaler was prescribed an additional 2306 times when compared to the same period the year before: a 152% increase (see Table 3). 

## 4. Discussion

### 4.1. PM_2.5_ and Prescriptions

The means and median PM_2.5_ concentrations were significantly different in December 2019 and January 2020 (Black Summer bushfires) compared to the corresponding control periods. The means and median PM_2.5_ concentrations were not significantly different between November 2019 and November 2018; thus, November 2019 was not significantly impacted by bushfire smoke; therefore, we excluded November when comparing the number of medicines dispensed between the bushfire and control periods.

Acute exposure to PM_2.5_ can trigger an asthma attack and increase symptoms of asthma (shortness of breath, cough, wheezing, chest tightness, and closure of the bronchial airways) [5,29]. Short acting beta-agonists are prescribed to relieve the acute symptoms of asthma and chronic obstructive pulmonary disease (COPD). Derived from previous published evidence that particulate matters are associated with increased salbutamol prescribing and patient use [20,21], we hypothesised that during the bushfire season more asthma medicines would be dispensed across the drug classes. 

When viewed categorically by drug class, our findings revealed an increase in the number of asthma prescriptions dispensed across all relevant drug classes (SABAs, LABAs, OCS, SABAs + ICS, and ICS) during the bushfire affected season. This includes relievers, preventers, and combination products. It should be noted that when looking at active ingredients individually, inconsistent with all other active ingredients salmeterol and budesonide were prescribed fewer times during the bushfire period, when compared to the control period. The greatest increase in dispensed prescriptions was for the combined preventer and reliever inhalers.

While the monthly mean PM_2.5_ was lower in January 2020 than in the month prior, December 2019, the number of dispensed prescriptions was highest in January 2020. This may be because individuals had medicines on hand that could be used before needing an original or refill prescription to be dispensed. It should be noted that the date a prescription is written is not always the date that the prescription is dispensed at a pharmacy. However, dispensing always comes after the prescription is issued.

In Australia, salbutamol is available both on prescription and as a Schedule 3 (pharmacist only) medicine. That is, it is available over the counter on recommendation from a pharmacist. Schedule 3 sales are private sales and are not captured in PBS data. Therefore, those individuals who had an asthma action plan, who presented to a pharmacy in the ACT may have been recommended salbutamol by their pharmacist. In such cases salbutamol can be provided to those in need, without it being recorded in the national dataset and subsequently was not analysed in our results. Future studies should explore the trends in provision of Schedule 3 salbutamol during periods of high PM_2.5_ concentrations, and the corresponding poor air quality. This would enable a more complete picture of the supply of asthma medicines during a bushfire and help inform stock holdings, to reduce short supplies.

While not a first-line pharmacotherapy for acute asthma, budesonide + formoterol (ICS + LABA), can also be used for both daily maintenance (as a preventor) and when required as a reliever medication for some patients [30]. Our study showed that this combination product had the greatest increase in dispensation, compared to all other combination products with active ingredients from similar drug classes. Perhaps this increase is due to its dual indication [21]. 

This study showed a 16% increase in the total number of oral corticosteroid prescriptions dispensed during the bushfire affected period. Oral corticosteroids can be used to treat acute asthma exacerbations by controlling the underlying inflammation, but they are also used in a wide array of other inflammatory conditions such as rheumatoid arthritis and gout [29,31]. As we could not control for these variables, a more detailed prescription dataset, which contains patients’ information, is needed to determine the association between asthma-related oral corticosteroid prescriptions and PM_2.5_.

Our study showed that bushfire-related smoke may be associated with the number of asthma medicine prescriptions that are dispensed in a defined geographic area. This presents data that pharmacists can use to pre-empt an increase in the supply and demand for asthma medicines. Bushfires present many logistical challenges for pharmacists when supplying medicines to their community [32]. Asthma, by its very nature, can be life threatening, and thereby it is imperative that pharmacists and manufacturers are cognisant that the poor air quality generated by bushfires, also drives up the need and demand for asthma medicines. This paper helps quantify what active ingredients are sought by the public, and at what increased magnitude. This awareness may help to ensure that more medicines are ordered and are on hand when patients present with prescriptions to be filled. 

There are many possible explanations for increased prescriptions during a bushfire season: (1) Patients may experience an increase in the number and severity of asthma symptoms, require more medications and step-up treatments [33]. (2) Concentrated smoke increases the incidence of new-onset asthma and likely drives up medication need. (3) Patients can request multiple prescriptions to be dispensed at one time; it is known that many Australians stockpile during natural disasters to mitigate the effect of stock shortages. Stockpiling of medicines does not reflect a worsening or exacerbation of asthma symptoms. (4) Prescribers may have given out more prescriptions as part of a preventative strategy for their patients. Many published studies indicate that high particulate matter concentrations and reduced air quality are associated with worsening asthma [13,18]. (5) Patients who lived near or within the bushfire areas might have had their medicines destroyed by fire and now require additional supplies [34]. 

Although this study cannot be used as direct evidence for an increased exacerbation of asthma when PM_2.5_ is high it can be used to inform the pharmacy profession, and others involved in medicines supply, about how to prepare stock during a bushfire season to ensure the continuous supply of lifesaving medications.

### 4.2. Confounding Factors

Based on the population growth projection from the ACT treasury, the population in the ACT from Dec 2018 to Jan 2020 increased by no more than 2% [35]. Furthermore, the prevalence of patients with asthma is around 11% of the total population based on data from the Australian Bureau of statistics [36]. We, therefore, estimated that there was around a 0.2% increase in patients with asthma and the demand for the medications from Dec 2018 to Jan 2020. Looking at the dispensing count for the SABA drug class, the number increased from January 2019 to January 2020 would be from 2043 to 2047 units of medication. Therefore, the effect of population growth was minimal and negligible in our analysis. 

We also considered the potential role of panic buying and stockpiling of medicines due to the COVID-19 pandemic (especially for salbutamol) to be another confounding factor [37]. It was reported that in late February 2020, a Canberra-associated COVID-19 case was identified in Sydney. Furthermore, the first COVID-19 case identified in Canberra was on 12 March 2020 [38]. The stockpiling and panic buying events started from around late February to early March 2020 [39]. Thus, we can safely conclude that COVID-19 itself was unlikely to affect medicine use in our study period or immediately prior. The COVID-19 pandemic also informed the use of the control period; therefore, we used the period outside the pandemic to help remove this lurking variable. 

### 4.3. Study Limitations

While inclusive of prescriptions dispensed across all pharmacies in the ACT, the obtained PBS statistics can only represent PBS and RPBS subsidised medications prescribed by a prescriber and dispensed in a pharmacy to eligible pharmacy patients. Therefore, our sample does not include medicines supplied to private patients, nor the salbutamol and terbutaline provided as a Pharmacist Only Schedule 3 medicine. A complete picture of the medication supplied may be obtained by gathering information directly from the pharmacies. Furthermore, we could only obtain monthly PBS data, not daily prescribing data; therefore, drawing a correlation between daily air quality and daily dispensed prescriptions could not be achieved.

Another limitation of our study is that we only examined data from the Civic monitoring station in Canberra. Thus, we might have underestimated the PM_2.5_ concentrations for the whole ACT region. In addition, as we did not fill in the missing data points for December 2018, this has likely led to an underestimation of the PM_2.5_ concentrations for that month.

## 5. Conclusions

In conclusion, bushfires can decrease air quality by increasing airborne PM_2.5_ concentrations. This study quantified and showed an increase in the number of asthma medicines dispensed during a bushfire season. All drug classes increased in dispensing count. The most significant increase was for the inhaled combination products. Our findings may provide insight to help manage medication stock and ensure the accessibility of those medications for the public during bushfire seasons.

## Figures and Tables

**Table 1:** Common asthma medications and their associated Pharmaceutical Benefits Scheme (PBS) item codes [25].

Class	Medication Name	PBS Item Codes (Obtained from PBS Website)
Short-acting beta agonist (SABA)	Salbutamol	8354Q, 11125M, 11130T, 2001H, 3497C, 2000G, 3496B, 1103C, 11088N, 11095Y, 3495Y, 8288F
	Terbutaline	2817G, 1034K,
Long-acting beta agonist (LABA)	Salmeterol	8141L
Long-acting beta agonists combined with inhaled corticosteroids (LABA + ICS)	Salmeterol + fluticasone	8519J, 8431R, 8517G, 8430Q, 8432T, 8518H,
	Budesonide + formoterol	11273H, 12029D, 12041R, 12093L, 8625Y, 10024N, 12100W, 10015D, 12042T, 12089G, 12101X, 8796Y, 10018G,12082X,11301T,8750M
	Fluticasone + formoterol	10007Q, 10008R, 2827T
	Vilanterol + fluticasone	11129R, 11124L
Inhaled corticosteroids (ICS)	Beclometasone	8407L, 8408M, 8409N, 8406K
	Budesonide	2070Y, 2065Q, 2072C, 2071B, 2066R
	Fluticasone	8346G, 8345F, 8148W, 8516F, 8147T, 8149X
Oral corticosteroids (OCS)	Prednisolone	1916W, 1917X, 3152X, 8285C
	Prednisone	1934T, 1935W, 1936X

**Table 2:** PM_2.5_ concentrations (µg/m^3^).

Month/Year	N (Days in the Month)	Bushfire Affected	Mean	Median (IQR)	Range
November 2018	30	No	5.317	4.8 (3.625–6.325)	1.5–13.9
December 2018	28	No	8.3	6.4 (5.025–9.2)	1.6–35
January 2019	31	No	8.597	8.5 (5.6–11.3)	2.1–14.8
November 2019	30	Yes	11.037	6.25	2.1–64.0
December 2019	31	Yes	68.471	56.5 (25.3–90.7)	2.6–239.2
January/2020	31	Yes	108.745	34.1 (19.8–91.3)	4.2–859

**Table 3:** Total prescription counts by drug. SABA: short-acting beta agonist; ICS: inhaled corticosteroids; OCS: Oral corticosteroids; LABA: long-acting beta agonists.

Drug Class	Drug	December 2018	January 2019	December 2019	January 2020	% Increase
SABA	Salbutamol	2548	1964	2595	3525	136
	Terbutaline	100	79	89	117	115
LABA	Formoterol	2729	2085	2442	2420	101
	Salmeterol	39	26	21	29	77
OCS	Prednisolone	1475	1279	1467	1741	116
	Prednisone	361	302	360	401	115
SABA + ICS	Salmeterol + Fluticasone	3111	2692	2883	4314	124
	Budesonide + formoterol	2335	2111	2691	4061	152
	Fluticasone + formoterol	150	143	131	237	126
	Vilanterol + fluticasone	1118	1039	1331	1813	146
ICS	Beclomethasone	17	18	18	36	154
	Budesonide	160	119	111	162	98
	Fluticasone	448	378	511	828	162
	Ciclesonide	123	86	94	131	108

## Data Availability

The research data (i.e., the measurements of the particulate matter of the air and the asthma medicines prescription dispensing data were obtained from publicly available sources (Australian Government) referenced in the text. All the research data have been shared through the Supplementary Materials mentioned above. No new data were generated in this study.

## Supplementary Materials

The following supporting information can be downloaded at https://www.mdpi.com/article/10.3390/healthcare12040428/s1.
